# The α*-*amylase and α-glucosidase inhibitory activities of the dichloromethane extracts and constituents of *Ferulago bracteata* roots

**DOI:** 10.1080/13880209.2017.1414857

**Published:** 2017-12-12

**Authors:** Songül Karakaya, Sefa Gözcü, Zühal Güvenalp, Hilal Özbek, Hafize Yuca, Benan Dursunoğlu, Cavit Kazaz, Ceyda Sibel Kılıç

**Affiliations:** a Department of Pharmacognosy, Faculty of Pharmacy, Atatürk University, Erzurum, Turkey;; b Department of Pharmacognosy, Faculty of Pharmacy, Erzincan University, Erzincan, Turkey;; c Department of Chemistry, Faculty of Science, Atatürk University, Erzurum, Turkey;; d Department of Pharmaceutical Botany, Faculty of Pharmacy, Ankara University, Ankara, Turkey

**Keywords:** Apiaceae, coumarin, peucedanol-2′-benzoate, felamidin, suberosin

## Abstract

**Context:***Ferulago* (Apiaceae) species have been used since ancient times for the treatment of intestinal worms, hemorrhoids, and as a tonic, digestive, aphrodisiac, or sedative, as well as in salads or as a spice due to their special odors.

**Objectives**: This study reports the α-amylase and α-glucosidase inhibitory activities of dichloromethane extract and bioactive compounds isolated from *Ferulago bracteata* Boiss. & Hausskn. roots.

**Materials and methods:** The isolated compounds obtained from dichloromethane extract of *Ferulago bracteata* roots through bioassay-guided fractionation and isolation process were evaluated for their *in vitro* α-amylase and α-glucosidase inhibitory activities at 5000–400 µg/mL concentrations. Compound structures were elucidated by detailed analyses (NMR and MS).

**Results:** A new coumarin, peucedanol-2′-benzoate (**1**), along with nine known ones, osthole (**2**), imperatorin (**3**), bergapten (**4**), prantschimgin (**5**), grandivitinol (**6**), suberosin (**7**), xanthotoxin (**8**), felamidin (**9**), umbelliferone (**10**), and a sterol mixture consisted of stigmasterol (**11**), β-sitosterol (**12**) was isolated from the roots of *F. bracteata*. Felamidin and suberosin showed significant α-glucosidase inhibitory activity (IC_50_ 0.42 and 0.89 mg/mL, respectively) when compared to the reference standard acarbose (IC_50_ 4.95 mg/mL). However, none of the tested extracts were found to be active on α-amylase inhibition.

**Discussion and conclusions:** The present study demonstrated that among the compounds isolated from CH_2_Cl_2_ fraction of *F. bracteata* roots, coumarins were determined as the main chemical constituents of this fraction. This is the first report on isolation and characterization of the bioactive compounds from root extracts of *F. bracteata* and on their α-amylase and α-glucosidase inhibitory activities.

## Introduction

*Ferulago* W. Koch. (Apiaceae) is represented by approximately 50 taxa throughout the world and 35 taxa in Turkey, 18 of which are endemic. Therefore, Anatolia is considered to be the gene center of this genus (Güner et al. 2012). *Ferulago bracteata* Boiss. & Hausskn. is also an endemic perennial species, grown only in Gaziantep, Southeastern Anatolia, Turkey (Peşmen 1972; Troia et al. 2012).

*Ferulago* species have been used in folk medicine for their aphrodisiac, digestive, tonic, sedative, antiseptic, carminative, and vermifuge properties as well as for the treatment of hemorrhoids, ulcers, snake bites, spleen diseases and headaches (Boulus 1983; Demetzos et al. 2000). Other than medicinal usage, they have been consumed as salad or spice due to their special odor, also as food for goats and deer (Erdurak 2003).

Previous phytochemical studies indicated that coumarins are the most common metabolites on *Ferulago* species (Jimenez et al. 2000). Coumarins have various biological activities such as anticancer (Lee et al. 2011; Luo et al. 2011), anti-inflammatory (Kwon et al. 2011; Huang et al. 2012), anticoagulant (Aoyama et al. 1992; Hirsh et al. 2001), antiadipogenic (Shin et al. 2010), antitubercular (Chiang et al. 2010), antihyperglycemic (Fort et al. 2000; Tchamadeu et al. 2010), antiviral (Venugopala et al. 2013), antifungal (Chou et al. 2007; Wang et al. 2009), antibacterial (Rosselli et al. 2009), antihypertensive (Crichton & Waterman, 1978; Gantimur et al. 1986), anticonvulsant (Luszczki et al. 2009), antioxidant (Kim et al. 2008; Basile et al. 2009), neuroprotective (Wang et al. 2012), and antidiabetic (Marles & Farnsworth 1995; Patel et al. 2012). This is the first report of isolation and structure elucidation study on the roots of *F. bracteata* to afford a new coumarin, peucedanol-2′-benzoate (**1**), along with nine known ones (**2–10**) and a sterol mixture (**11–12**). The α-amylase and α-glucosidase inhibitory activities of isolated coumarins were also evaluated. Coumarins may be a potential source of new antidiabetic agents and may also be used by peripheral tissues by improving insulin resistance and increasing glucose uptake (Zhang et al. 2017). Peucedanol 7-*O*-β-d-glucopyranoside (Lee et al. 2004), coumarin (1,2-benzopyrone) (Pari & Rajarajeswari 2009) umbelliferone (Ramesh & Pugalendi 2005), imperatorin (Adebajo et al. 2009), psoralen, 5-methoxypsoralen, 8-methoxypsoralen, isooxypeucedanin, pabulenol, oxypeucedanin methanolate, oxypeucedanin hydrate (Shalaby et al. 2014), isobergapten, pimpinellin, isopimpinellin, sphondin, scopoletin, phellopterin, byakangelicin and daucosterol (Zhang et al. 2017) were isolated from various plants belonging to the Apiaceae family and were found to be antidiabetic. So, this may be a significant approach in the treatment of type 2 diabetes. This study is a first report on the isolation and characterization of the bioactive compounds from root extracts of *F. bracteata* and also reports α-amylase and α-glucosidase inhibitory activities of this species.

## Materials and methods

### General

NMR spectra were recorded on a Varian Mercury Plus at 400 MHz for ^1 ^H NMR and 100 MHz for ^13 ^C NMR by using TMS as the internal standard. The solvents were CDCl_3_. HR-ESI-MS was performed on Agilent 6530 Accurate- Mass Q-TOF LC/MS. UV spectra were measured using Thermo Scientific Multiskan GO microplate and cuvette spectrophotometer. IR spectra were run on a Bruker VERTEX 70v FT-IR Spectrophotometer. Column chromatographies were performed on Silica gel 60 (0.063–0.200 mm, Merck) and Sephadex LH-20 (Fluka). TLC was carried out on pre-coated Kieselgel 60 F_254_ aluminum sheets (Merck).

### Plant material

The roots of *F. bracteata* were collected in July 2014 from Gaziantep (C6 Gaziantep: Nurdağı Antep road 22. km, calcareous cliff rocks, 1642 m east of Turkey, Coordinates: 37° 11′ 226 N, 36° 57′ 792 E) and identified by Prof. Dr. Hayri Duman, a plant taxonomist in the Department of Biological Sciences, Faculty of Art and Sciences, Gazi University. A voucher specimen (No AEF 26676) is stored in the Herbarium of Faculty of Pharmacy, Ankara University, Turkey.

### Extraction and isolation

Air-dried roots of *F. bracteata* (450 g) were powdered and macerated three times with methanol for 8 h in a water bath not exceeding 45 °C (4 × 2 L) using a mechanical mixer at 300 rpm. Combined extracts were filtered and concentrated to dryness (60.94 g), then dispersed in methanol:water (1:9) and fractionated four times with 400 mL of dichloromethane, ethyl acetate, and *n*-butanol, respectively. Each fraction was concentrated to dryness, and 17.96 g dichloromethane, 2.44 g ethyl acetate, and 14.98 g of *n*-butanol fractions were obtained. Finally, 23.55 g of aqueous fraction remained. The roots (50 g) were grounded and macerated with 500 mL of distilled water for 8 h/3 days at 30 to 35 °C. This material was filtered and lyophilized to give aqueous extracts of 3.99 g roots.

As a result of the bioguided fractionation study, the effective dichloromethane extract was first submitted to a silica gel column (Merck 737, 70–230 Mesh) and eluted with a gradient of *n-*hexane:ethyl acetate (100:0 → 0:100, *v/v*) and ethyl acetate:methanol (100:0 → 0:100, *v/v*), and eight fractions (Fr. A–H) were obtained. Fr. A was subjected to a silica gel column, which was eluted with a mixture of *n-*hexane:ethyl acetate (95:5) and compounds **11** and **12** were obtained as a mixture (217 mg). Repetitive silica gel column chromatography with *n*-hexane:ethyl acetate (90:10 and 95:5) solvent system on Fr. B gave compound **2** (220 mg). Fr. C was applied to silica gel column eluting with *n*-hexane:ethyl acetate (85:15) and Sephadex LH-20 column eluting with ethyl acetate to give compounds **3** (125 mg) and **4** (130 mg). Eluting with *n*-hexane-ethyl acetate (90:10) over silica gel column of Fr. D gave compound **5** (400 mg) and Fr. E gave compounds **1** (320 mg), **6** (150 mg), and **7** (330 mg). Fr. F was eluted with 25% ethyl acetate in *n*-hexane and rechromatographed with 25% ethyl acetate in *n*-hexane on silica gel column to obtain compound **8** (110 mg). Fr. G was fractioned by column chromatography over silica gel using *n*-hexane:ethyl acetate mixtures (70:30 and 90:10) consecutively to obtain compound **9** (325 mg). Fr. H was submitted on a silica gel column using *n*-hexane:ethyl acetate (65:35) and the resulting fraction was chromatographed on silica gel column using *n*-hexane:ethyl acetate (90:10) to obtain compound **10**.

### Peucedanol-2'-benzoate (1)

White powder; IR *ν*_max_ (KBr) cm^−1^: 1702, 1623, 1565; UV *λ*_max_ (CH_2_Cl_2_) nm (log *ɛ*): 350 (4.20); ^1 ^H NMR (400 MHz, CDCl_3_) and ^13 ^C NMR (100 MHz, CDCl_3_) spectroscopic data, see Table 1; HR-ESI-MS at *m/z* 367.1999 [M-H]^+^ (Calculated for C_21_H_19_O_6_ 367.1181).

**Table 1.** ^1 ^H NMR (400 MHz) and ^13 ^C NMR (100 MHz) data of **1** in CDCl_3_.

No	*δ*_H_ (*J* in Hz)	*δ*_C_
2		161.38
3	6.26 (d, 9.4)	112.37
4	7.64 (d, 9.4)	143.62
5	7.28 (s)	123.22
6		124.55
7		163.49
8	6.80 (s)	98.01
9		155.84
10		112.71
1′	3.38 (m)	29.67
2′	5.16 (dd, 9.2, 7.3)	89.12
3′		82.93
4′	1.72 (s)	22.16
5′	1.71 (s)	21.38
1′′		165.40
2′′		131.04
3′′	7.73 (m)	129.39
4′′	7.32 (m)	128.27
5′′	7.51 (m)	132.86
6′′	7.32 (m)	128.27
7′′	7.73 (m)	129.39

### Antidiabetic activity

#### α-Amylase inhibitory activity

α-Amylase inhibitory activity was established in accordance with the reported method (Nampoothiri et al. 2011) with slight modifications. One percent starch solution (100 µL) in 20 mM sodium phosphate buffer (pH 6.9 with 6 mM sodium chloride) and sample solutions (100 µL) were incubated at 25 °C for 10 min in 24-well microplate. After incubation, 100 µL α-amylase solution (0.5 mg/mL) was added to each well and the reaction mixtures were incubated at 25 °C for 10 min. In order to stop the reaction after the incubation, dinitrosalicylic acid color reagent (200 µL) was added and then the microplate was incubated in a boiling water bath for 5 min and cooled at room temperature. 50 µL was taken from each well and then was added to 96-well microplate. The reaction mixture was diluted by adding 200 µL distilled water and absorbance was measured at 540 nm. Each assay for all samples was carried out in triplicate. Percentage inhibitions of all samples were calculated using the equation at following:

$$Inhibition\;\left(\%\right)=\left(1-\frac{{\Delta A}_{\text{sample}}\;}{{\Delta A}_{control}}\right)\times 100$$

#### α-Glucosidase inhibitory activity

α-Glucosidase inhibitory activity was established by using a 96-well microtiter plate in accordance with the described method (Tao et al. 2013) with slight modifications. *p*-Nitro-phenyl-α-d-glucopyranoside (*p*-NPG) was used as the substrate and was prepared in 0.1 M potassium phosphate buffer (pH 6.8). α-Glucosidase (0.1 Unit/mL, enzyme solution) was dissolved in the same buffer. Samples were dissolved in dimethyl sulfoxide (DMSO) and all samples (20 µL) together with the enzyme solution (20 µL) were mixed in the plate. Afterwards, the substrate (40 µL) was added for initiation of the reaction and the mixture was incubated at 37 °C for 40 min. After incubation is complete, 0.2 M sodium carbonate (80 µL) in phosphate buffer (pH 6.8) was added to all wells in order to quench the reaction. The amount of released *p*-nitrophenol (pNP) was measured at 405 nm using a 96-well microplate reader. Each assay for all samples was carried out in triplicate. Percentage inhibition of all samples was calculated using the following equation:

$$Inhibition\;\left(\%\right)=\left(1-\frac{{\Delta A}_{\text{sample}}\;}{{\Delta A}_{control}}\right)\times 100$$

### Statistical analysis

All results are expressed as mean ± SE and differences between means were statistically analyzed using one-way analysis of ANOVA followed by Bonferroni’s complementary analysis, with *p* < 0.05 considered to indicate statistical significance.

## Results and discussion

**Figure 1.**
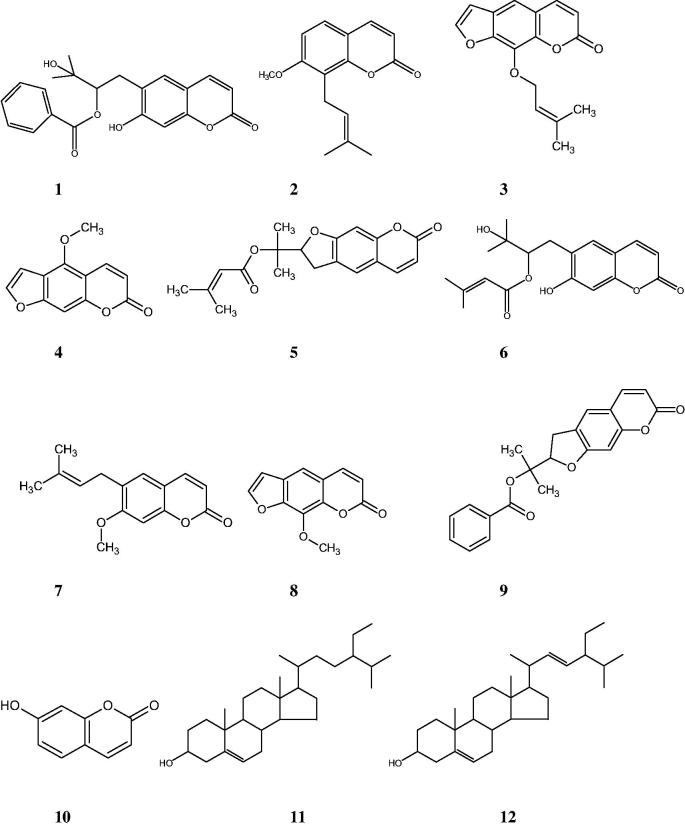
Chemical structures of compounds **1–12**.

Methanol extract of the roots of *F. bracteata* was fractionated using solvents with different polarities (*n*-hexane, dichloromethane, ethyl acetate, and *n*-butanol) and the obtained fractions were evaluated for their α-amylase and α-glucosidase inhibitory activities. The active dichloromethane extract was subjected to column chromatography over silica gel and Sephadex LH-20. As a result, a new coumarin, peucedanol-2′-benzoate (**1**), together with nine known ones, osthole (**2**) (Sajjadi et al. 2009), imperatorin (**3**) (Muller et al. 2004), bergapten (**4**) (Stevenson et al. 2003), prantschimgin (**5**) (Sajjadi et al. 2015), grandivitinol (**6**) (Abyshev et al. 1977), suberosin (**7**) (Tabanca et al. 2016), xanthotoxin (**8**) (Stevenson et al. 2003), felamidin (Kilic et al. 2006) (**9**), umbelliferone (**10**) (Singh et al. 2010), and a sterol mixture consisted of stigmasterol (**11**), β-sitosterol (**12**) (Woldeyes et al. 2012) (Figure 1) were isolated and identified.

**Figure 2.**
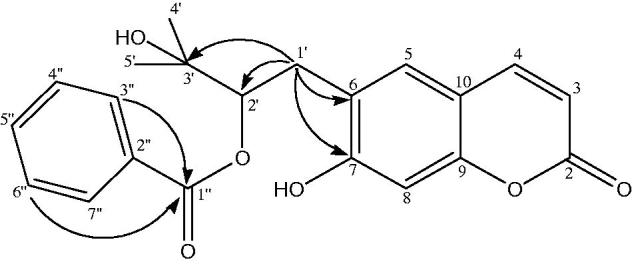
Significant HMBC (→) correlations of compound **1**.

Peucedanol-2′-benzoate (**1**) was isolated as a white powder with the molecular formula of C_21_H_20_O_6_ as determined by the HR-ESI-MS at *m/z* 367.1999 [M − H]^+^ (Calcd for C_21_H_19_O_6_ 367.1181). The IR spectrum of **1** showed absorption bands for C = O groups (1702 cm^−1^) and -CH = CH- bonds (1623, 1565 cm^−1^). The ^1 ^H NMR spectrum of compound **1** displayed two AB type system protons at *δ*_H_ 6.26 and 7.64 (each 1 H, d, J = 9.4 Hz) which was attributed to the H-3 and H-4 protons of the coumarin nucleus. The two single aromatic proton signals at *δ*_H_ 7.28 and 6.80 were assigned to H-5 and H-8 protons. The ^13 ^C NMR spectrum revealed the presence of 9 carbons resonances including four methine [*δ*_C_ 112.37 (C-3), 143.62 (C-4), 123.22 (C-5), 98.01 (C-8)], three oxygenated quaternary [*δ*_C_ 161.38 (C-2), 163.49 (C-7), 155.84 (C-9)], and two non-oxygenated quaternary carbons [*δ*_C_ 124.55 (C-6), 112.71 (C-10)] for coumarin skeleton. Two tertiary methyl groups at *δ*_H_ 1.72 (3 H, *s*, H-4′), 1.71 (3 H, *s*, H-5′) and at *δ*_C_ 22.16 (C-4′), 21.38 (C-5′) with the hydroxyl group; an oxygenated methine at *δ*_H_ 5.16 (1 H, dd, J = 9.2/7.3 Hz, H-2′) and at *δ*_C_ 89.12 (C-2′); and a methylene at *δ*_H_ 3.38 (2 H, m, H-1′) and at *δ*_C_ 29.67 (C-1′) confirmed the 2′,3′-dihydroxy-3′-methyl butyl moiety. HMBC correlation (Figure 2) between H-1′ (*δ*_H_ 3.38) and C-6 (*δ*_C_ 124.55) suggested that it was attached to C-6 position. Characteristic signals of a benzoyl moiety were also exhibited, including a pair of 2 H at *δ*_H_ 7.73 (H-3″, H-7″) and 7.32 (H-4″, H-6″) and 1 H at 7.51 (H-5″) in the ^1 ^H NMR spectrum and aromatic carbons at *δ*_C_ 131.04 (C-2″), 129.39 (C-3″, C-7″), 128.27 (C-4″, C-6″), 132.86 (C-5″) with a carbonyl carbon at *δ*_C_ 165.40 (C-1″) in the ^13 ^C NMR spectrum. The linkage of the benzoyl group to the 2′,3′-dihydroxy-3′-methyl butyl moiety was deduced from the downfield shifted signal of H-2′ (*δ*_H_ 5.16) and C-2′ (*δ*_C_ 89.12). Thus, the structure of the compound **1** was characterized as peucedanol-2′-benzoate.

**Figure 3.**
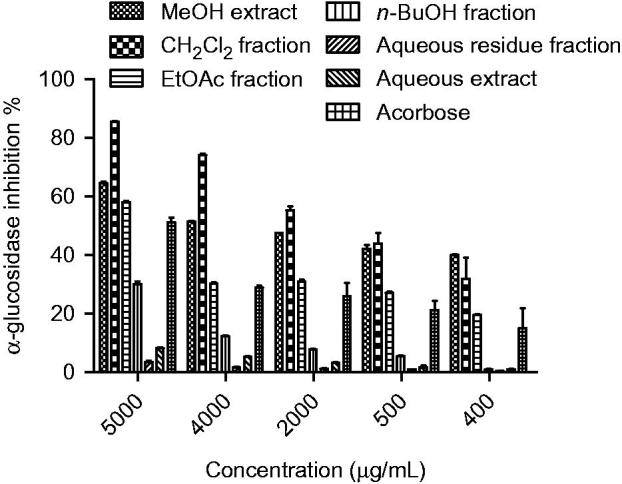
*α*-Glucosidase inhibition (%) of extracts and fractions of roots from *Ferulago bracteata* at different concentrations. The different extracts and fractions were compared with acarbose and *p* < 0.05 (p = 0.0004).

**Figure 4.**
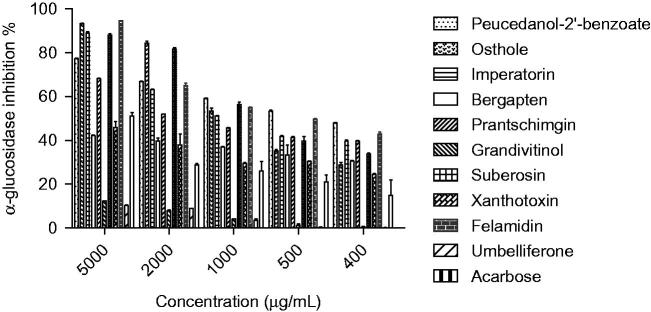
*α*-Glucosidase inhibition effects (%) of compounds **1–10**. The isolated coumarins were compared with acarbose and *p* <0.05 (p = 0.0437).

The extracts and compounds **1–10**, obtained via bioassay-guided fractionation and isolation process, were evaluated for their *in vitro* α-amylase and α-glucosidase inhibitory activities. The IC_50_ values and inhibitory effects (%) were given in Table 2 and Figure (3,4). Acarbose was used as a reference standard for both assays. Dichloromethane extract showed significant activity against α-glucosidase (IC_50_ 0.95 mg/mL) and among the tested compounds felamidin (IC_50_ 0.42 mg/mL) possessed the best inhibitory activity which were more potent than acarbose (IC_50_ 4.95 mg/mL). Suberosin, osthole, imperatorin, prantschimgin, peucedanol-2′ benzoate also showed α-glucosidase inhibitory activity (IC_50_ 0.89, 0.95, 1.23, 1.86, 3.24 mg/mL, respectively) which had lower effect than felamidin but stronger than acarbose. Among the tested compounds bergapten, xanthotoxin, and umbelliferone (IC_50_ 6.12, 5.38, and 9.32 mg/mL, respectively) showed lower activity than reference compound acarbose. Grandivitinol (IC_50_ 20.01 mg/mL) possessed the worst inhibitory activity. However, none of the extracts showed meaningful α-amylase inhibitory activity, while acarbose indicated 82.28% inhibition at a concentration of 1 mg/mL. These results indicate that felamidin was 11 times more effective than acarbose against α-glucosidase.

**Table 2.** *α*-Glucosidase inhibitory activities of extracts, fractions and compounds **1–10**.

Extracts/Fractions/Compounds	Concentration (µg/mL)	*α*-Glucosidase inhibition (%)	IC_50_ value (mg/mL)
Methanol extract	5000	64.44 ± 1.47	4.19
	2000	51.42 ± 0.53	
Dichloromethane fraction	5000	85.52 ± 0.38	0.95
	2000	74.19 ± 0.68	
Ethyl acetate fraction	5000	58.04 ± 0.76	4.20
	2000	30.24 ± 0.75	
*n*-Butanol fraction	5000	30.05 ± 1.63	8.35
	2000	12.18 ± 0.35	
Aqueous residue fraction	5000	3.87 ± 1.28	ND
	2000	1.06 ± 0.75	
Aqueous extract	5000	8.55 ± 1.92	ND
	2000	5. 48 ± 1.23	
Peucedanol-2'-benzoate	5000	77.22 ± 0.44	3.24
	2000	66.86 ± 0.37	
Osthole	5000	93.31 ± 0.29	0.95
	2000	84.31 ± 1.72	
Imperatorin	5000	88.97 ± 0.88	1.23
	2000	63.07 ± 0.69	
Bergapten	5000	42.26 ± 0.43	6.12
	2000	39.71 ± 2.44	
Prantschimgin	5000	68.23 ± 0.39	1.86
	2000	51.95 ± 0.01	
Grandivitinol	5000	12.30 ± 0.36	20.01
	2000	7.84 ± 0.71	
Suberosin	5000	87.95 ± 1.07	0.89
	2000	81.58 ± 1.15	
Xanthotoxin	5000	45.76 ± 4.82	5.38
	2000	37.95 ± 8.64	
Felamidin	5000	94.57 ± 0.05	0.42
	2000	64.93 ± 2.06	
Umbelliferone	5000	10.27 ± 0.49	9.32
	2000	8.84 ± 0.02	
Acarbose	5000	50.81 ± 2.51	4.95
	2000	29.43 ± 1.71	

Data are means ± SD of three replicates in each group.

To our knowledge, no previous studies have been reported on α-glucosidase and α-amylase inhibitory activities of *F. bracteata* and the isolated coumarins prantschimgin, peucedanol-2′-benzoate, felamidin, grandivitinol, and suberosin. Also, this is the first report on the phytochemical analysis of *F. bracteata*.

Our results are similar to the previous studies performed on related coumarins. Shalaby et al. (2014) found that imperatorin (at 1000 µg/mL α-glucosidase inhibition% was found to be 69.66 ± 3.67 and we found an inhibition of 88.97 ± 0.88% at a concentration of 5000 µg/mL) showed appreciable antidiabetic activity. Comparing these results with previous studies in which α-glucosidase IC_50_ value of umbelliferone was found to be 7.79 ± 0.11 μg/mL, we have found a higher inhibitory activity with 9.32 mg/mL (Ramith et al. 2014). Comparing these results with a previous study in which α-glucosidase IC_50_ value of umbelliferone was 0.547 mg/mL at 0.5 mg/mL, the inhibitory activity that we found was higher (Ayyasamy & Rajamanickam 2015). Luo et al. (2012) reported that α-glucosidase inhibitory activity of bergapten, xanthotoxin, and imperatorin has been too low to compare to that of acarbose. In our study, it was determined that bergapten and xanthotoxin showed similar effects, however, imperatorin was more effective than acarbose. In spite of the advances in biomedical science and the introduction of new treatment ways, diabetes mellitus has been a major cause of end-stage renal disease, new-onset blindness, and cardiovascular diseases, all of which cause excess mortality and morbidity in people with diabetes (Islam et al. 2013). Medical herbs are generally rich in phenolic compounds, such as phenolic acids, flavonoids, tannins, stilbenes, lignans, coumarins, and lignins (Celik et al. 2013). One of the therapeutic approach for treating diabetes is to reduce the postprandial hyperglycemia and this is made by delaying the absorption of glucose via the inhibition of the carbohydrate-hydrolyzing enzyme α-glucosidase in the digestive tract. Inhibitors of intestinal α-glucosidase have been utilized in the treatment of non-insulin-dependent diabetes mellitus and represented at the large ratio of antidiabetic drug market (Yin et al. 2008). The tested extracts and isolated compounds are rich in phenolic compounds, as well as in coumarins, which may contribute to its *in vitro* antidiabetic effect. In addition, this study suggests that the glucose-lowering effect of these plants can be due, at least in part, to the inhibition of α-glucosidase. Also, investigations are warranted to define the active principles and elucidate other possible mechanism(s) of action. Natural products are still considered as potential sources for drug exploration and play a significant role in drug development programs. Furthermore, many medicinal plants could be rich sources of bioactive chemicals that are importantly free from undesired adverse effects and show powerful pharmacological activities.

## Conclusions

In conclusion, the present study demonstrated that among the compounds isolated from CH_2_Cl_2_ fraction of *Ferulago bracteata* roots, coumarins were determined the main chemical constituents of this fraction. The most potent compounds found were felamidin and suberosin, respectively. Thus, *F. bracteata* along with its isolated compounds could be used for further studies on the development of novel preventive or therapeutic agents for the treatment of diabetes.
